# The Book World of Medicine and Science

**Published:** 1906-04-07

**Authors:** 


### The Book World of Medicine and Science.

Recent Advances in Physiology and Biochemistry. Edited by Leonard Hill, M.B., F.R.S. (London: Edward Arnold. 1906. Pp. 728. Price 18s. net.)

At the present day, with the great strides that the science of physiology is making, it is interesting and useful to have some of the latest work collected together and laid before us in book form, as is done in "Recent Advances in Physiology and Biochemistry." Dr. Hill is to be congratulated on the able and distinguished co-workers that he has secured to contribute articles to the book. Professor Benjamin Moore has written the chapter on biochemistry and the action of enzymes, subjects on which he has himself done a great deal of original work. Dr. Hill has taken for his share chapters on the atmosphere, on the effects of increased and diminished barometric pressure, reference being made especially to "mountain sickness" and "caisson disease," and also on the metabolism of fat and its bearing on obesity. Professor Macleod deals with the metabolism of carbohydrates, of purin bodies, and the subject of hæmolysins and allied bodies which are essentially physiological properties of normal blood serum. Dr. Pembrey enters into the subject of respiratory exchange in all its bearings, and also treats of internal secretion. Dr. Beddard has written the chapters on lymph formation and absorption, on the formation of urea, and the mechanism of the secretion of urine. At the end of each article sufficient references are given to enable the reader to find his way into the full literature on the subject. Throughout the book the subjects that have been chosen are those that have most bearing on practical medicine; thus the chapter on "hæmolysins and allied bodies" gives in detail all the recent work on hæmolysins, cytotoxins, præcipitins, agglutinins, and opsonins. The metabolism of the purin bodies is discussed in another chapter, and the methods of measuring the endogenous and exogenous moieties of purin excretion given. It is shown how the endogenous moiety is uninfluenced by the nature and amount of diet, provided always that it is purin free; and it is constant for the individual under the same physiological conditions. In the chapter on the metabolism of carbohydrates the primary cause of the hyperglycæmia in experimental diabetes and diabetes mellitus is ascribed to the withdrawal from the organism of some influence necessary for the utilisation of dextrose. The editor hopes, if this volume fulfils a want which he believes exists, to edit a subsequent volume, in which other subjects of equal interest and importance may be treated in a like manner. We therefore wish this book every success, so that in the near future we may have the chance of perusing a further selection of articles on physiology, which we are sure will be equally useful and instructive.

The Management of a Nerve Patient. By Alfred T. Schofield, M.D. (London: J. and A. Churchill, 1906. Pp. 267. 5s. net.)

Dr. Schofield is an industrious writer of popular works on matters psychological. He considers the medical profession guilty of a neglect of serious study of what he is pleased to term "mental therapeutics." In his latest monograph he attempts to describe in some details "the management of a functional nerve patient from the standpoint of a practical physician who is already imbued with a strong sense of the all-pervading rule of mind over matter." Although the work is dedicated to "my best nerve patient," and written in a style likely to attract the neurotic lay reader, the author is careful to point out that his work is primarily addressed to medical practitioners. Dr. Schofield would have us "concentrate our thoughts on the one great central force on the efficacy of which recovery in every disease ultimately depends, and that is 'Nature' or the *Vis Medicatrix, Naturæ*—in other words, the 'Unconscious Mind.'" What is meant by these comprehensive terms is explained at length in these pages in a peculiarly attractive, although, perhaps, not altogether convincing, manner. The present position of psycho-therapeutics is discussed in detail, and the author's view of its place in modern medicine is fully described. There is much that is of suggestive value and is likely to be of considerable service to the young practitioner inexperienced in the management of neurotic cases. Through ignorance and neglect many a patient is allowed to fall into the hands of quacks, and this book is intended to indicate the manner in which such tendency may be combated. Though we may not agree with the somewhat superficial presentation of many profound psychological problems, there is much that is praiseworthy and likely to be of practical use in these pages, and we would commend in particular the chapter on the "rest cure" and the sections dealing with the detailed individual treatment of neurasthenics.

Ellis's Demonstrations of Anatomy. Twelfth edition. Revised and edited by Christopher Addison. (London: Smith Elder and Company, pp. 851, with 306 engravings. Price 12s. 6d. net.)

A few years ago "Ellis's Demonstrations of Anatomy" was the text-book almost universally used by students in the dissecting-room; but lately it has been to a considerable extent displaced by works of more recent date and more modern arrangement. In the hope of restoring this book to its former popularity it has been extensively revised by Mr. Christopher Addison. The general style and character of the book has not been interfered with, but the matter has been entirely rearranged, and it now follows the ordinary course of dissection as taken by students, beginning with the simple anatomy of the upper and lower limbs and ending with the more complex parts of the head and neck and the organs contained therein. The descriptive matter has been elaborated and modernised, especially in those parts dealing with the viscera. The book is well indexed and illustrated—two features of paramount importance in a reference work of this kind. The illustrations of the bones showing in coloured outline the attachments of the muscles are especially useful.

